# Malaria Threatens to Bounce Back in Abergele District, Northeast Ethiopia: Five-Year Retrospective Trend Analysis from 2016-2020 in Nirak Health Center

**DOI:** 10.1155/2022/6388979

**Published:** 2022-06-07

**Authors:** Habtu Debash, Yonas Erkihun, Habtye Bisetegn

**Affiliations:** Department of Medical Laboratory Sciences, College of Medicine and Health Sciences, Wollo University, Dessie, Ethiopia

## Abstract

**Background:**

In Sub-Saharan African countries, malaria is a leading cause of morbidity and mortality. In Ethiopia, malaria is found in three-fourths of its land mass with more than 63 million people living in malaria endemic areas. Nowadays, Ethiopia is implementing a malaria elimination program with the goal of eliminating the disease by 2030. To assist this goal, the trends of malaria cases should be evaluated with a function of time in different areas of the country to develop area-specific evidence-based interventions. Therefore, this study was aimed at analysing a five year trend of malaria in Nirak Health Center, Abergele District, Northeast Ethiopia, from 2016 to 2020.

**Methods:**

A retrospective study was conducted at Nirak Health Center, Abergele District, Northeast Ethiopia from February to April 2021. Five-year (2016 to 2020) retrospective data were reviewed from the malaria registration laboratory logbook. The sociodemographic and malaria data were collected using a predesigned data collection sheet. Data were entered, cleaned, and analysed using SPSS version 26.

**Results:**

In the five-year period, a total of 19,433 malaria suspected patients were diagnosed by microscopic examination. Of these, 6,473 (33.3%) were positive for malaria parasites. Of the total confirmed cases, 5,900 (91.2%) were *P. falciparum* and 474 (7.2%) were *P. vivax*. Majority of the cases were males (62.2%) and in the age group of 15-45 years old (52.8%). The findings of this study showed an increasing trend in malaria cases in the past five years (2016-2020). The maximum number of confirmed malaria cases reported was in the year 2020, while the minimum number of confirmed malaria cases registered was in 2016. Regarding the seasonal distribution of malaria, the highest number of malaria cases (55.2%) was observed in Dry season (September to January) and also the least (15.9%) was observed in Autumn (March to May) replaced by the least (21.6%) was observed in Rainy season (June to August), that is, the major malaria transmission season in Ethiopia and the least (15.9%) was observed in autumn (March to May).

**Conclusion:**

The trends of malaria in Nirak Health Center showed steadily increasing from the year 2016–2020, and the predominant species isolated was *P. falciparum*. This showed that the malaria control and elimination strategy in the area were not properly implemented or failed to achieve its designed goal. Therefore, this finding alarms the local governments and other stack holders urgently to revise their intervention strategies and take action in the locality.

## 1. Background

Malaria is a vector-borne disease caused by Plasmodium protozoan parasites and transmitted to humans through the bite of an infected female *Anopheles mosquito* [1]. The most frequent species that cause malaria in humans are *Plasmodium falciparum* (*P. falciparum*) and *Plasmodium vivax* (*P. vivax*) [2]. Malaria is endemic in 85 countries worldwide with estimated 241 million cases in 2020 alone. It affects a wide range of people, most notably pregnant women and children, with an estimated 602,000 deaths in 2020. The African continent continues to bear a highest share of the global malaria burden. The region accounted for 95% of all malaria cases and 96% of all malaria deaths [3].

Malaria is one of the most serious public health problems in Sub-Saharan African countries including Ethiopia, limiting the country's production and development [3, 4]. The disease risk map covers around 68% of Ethiopia's total land area and 60% of the total population. The disease caused 1,206,892 confirmed and clinical malaria cases and 158 deaths in 2018 in Ethiopia. The proportion of *P. falciparum* was found to be 883,886 (69.2%) while *P. vivax* was found to be 181,964 (30.8%) [5].

In Ethiopia, malaria is among the top 10 communicable disease seen in health institutions, and its prevalence and transmission depend on a number of factors including temperature, rainfall, and altitude. In Ethiopia, the majority of malaria cases occur below 2000 meters above sea level, and it is less important in elevations above 2000. The country is known for unstable and seasonal malaria transmission which peaks from September to November, months that succeed wet and rainy summer or “keremit” season. Despite its low record in dry season, December to February, malaria case never touches zero and continues to build its second peak starting from the end of April to June, which are considered as the “belg” or short rainy season [6].

The national malaria control program (NMCP) is implementing different strategies including early detection of epidemics through appropriate surveillance, early diagnosis and treatment of cases, deployment of vector control methods, and community mobilization which aimed at awareness creation and environmental management to contain and control the disease [6]. In addition to the disease control and prevention program, the country is currently implementing a bold agenda of elimination program with the goal of eliminating the disease by 2030 [7, 8].

Malaria control measures that have been implemented worldwide yielded significant reduction of both disease morbidity and mortality, and Ethiopia is one of the countries that has made significant success [7]. Despite the documented success in bringing down the burden of the disease, malaria continues to be a public health concern in Ethiopia [9–11]. In Ethiopia, the disease's incidence, prevalence, and mortality rate all followed a similar pattern to that of global malaria case trend [6, 12, 13]. In contrary to the country's average which shows significant reductions both in morbidity and mortality, malaria problem remains concerning in some parts of the country such as Gambella, Benishangul, and Amhara regions. Thus, it commendable to develop interventions based on evidence in a given area, make well-informed decisions, and monitor the success of malaria control efforts. Moreover, continuous monitoring and evaluation of the ongoing interventions are needed to design counter measures in spots where the disease case trend shows upsurge.

In Abergele area, in particular, the general trend of malaria prevalence has not been examined. Analysing the malaria morbidity pattern in endemic areas would contribute to a better knowledge of disease transmission patterns and evaluating the effectiveness of malaria interventions for reducing disease burden in a given area. Laboratory registration books in health facilities are valuable sources of malaria data for epidemiological surveillance, planning malaria control programs, and evaluating the impact of health services at lower cost. Therefore, this study is aimed at examining malaria trends and transmission patterns during the previous five years at Nirak Health Center in Waghimra, Northeast Ethiopia, based on sex, age and season.

## 2. Materials and Methods

### 2.1. Study Area

This study was conducted in Nirak Health Center, Abergele district. It is located in the Waghimra zone, Amhara regional state, Ethiopia, at 780 kilometres north of Addis Ababa, capital city of Ethiopia. The district is located at 13°6′N 38°57′E, at an elevation of 1200 meters above sea level, and has a latitude and longitude of 13°6′N 38°57′E. The average yearly temperature and rainfall in the area are 26°C and 786 ml, respectively. Subsistence farming, livestock breeding, and fishing are the main occupations in the district. Water for mosquito breeding is available throughout the year because of rivers like Tekeze River. Therefore, this area is malarious, and Nirak Health Center is one of the four health centers in the district, which provides inpatient and outpatient services for more than 46,500 population.

### 2.2. Study Design, Sampling Method, and Period

A retrospective study was conducted to determine the five-year malaria trend from 2016 to 2020, by reviewing the laboratory registration logbooks from February to April 2021 at Nirak Health Center. The blood films examined over five years in the health center and fulfilling the inclusion criteria were recorded and analysed.

### 2.3. The Study Population and Data Collection

The study participants were all malaria suspected individuals who had complained of febrile illness at Nirak Health Center during the study period. Experienced medical laboratory professionals collected sociodemographic and laboratory data from patients' registration books using a predesigned data collection sheet.

At the time of data collection, malaria intervention activities that had been taken in each year to control malaria were identified using a well-prepared checklist from the chairperson of the district health office. The district health office's annual previous years malaria control and prevention strategy, as well as its achievements, were examined. We have also communicated with the head of the health center and local health extension workers. In the past years, malaria control and prevention activities were properly implemented by all stockholders. Community awareness raising regarding malaria transmission and control, early diagnosis and treatment, and the proper utilization of insecticide-treated nets (ITN) by the community were among the strategies used.

### 2.4. Statistical Analysis

Data were extracted from laboratory registration logbooks using well-prepared checklist. All data was checked for its completeness, then, the data was entered and analysed using SPSS 26 version software. Descriptive statistics were used to present the data and to evaluate malaria trends over years, months, and seasons. A chi-square test was used to describe the association of variables such as sex, age, month, year, and parasite species with malaria cases. The retrospective total malaria cases in the past five years (2016–2020) were summarized using figures and tables. *P* value less than 0.05 was considered as statistically significant.

## 3. Results

### 3.1. Trends in Malaria Cases from 2016-2020

During the last five years, from January 2016 to December 2020, a total of 19,433 patients who were clinically suspected of malaria have been examined for malaria parasites. Out of the total suspects, 11,111 (57.2%) were males, and 8,322 (42.8%) were females. Out of 19,433 tested patients, 6,473 (33.3%) were microscopically confirmed malaria cases. Of the total confirmed malaria cases, 4,023 (62.2%) were males and 2,450 (37.8%) were females. The predominant plasmodium species was *P. falciparum* 5,900 (91.2%), followed by *P. vivax* 474 (7.3%) and mixed infections (*P. falciparum* and *P. vivax*) 99 (1.5%) (Table 1).

**Figure 1:**
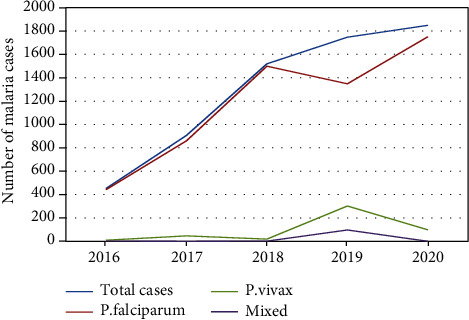
Five-year trend of malaria cases in Nirak Health Center, Abergele District, Northeast Ethiopia.

Results of the trend analysis showed an increasing trend of malaria cases in the past five years. The maximum number of malaria cases (1,849 (40.2%)) was reported in 2020. In contrast, the minimum number of confirmed malaria cases registered was 452 (15.8%) in 2016. Regarding the identified Plasmodium species, both species of Plasmodium were reported in each year. In the recent year 2019 to 2020, *P. falciparum* was increasing from 77.2% to 94.7%, but *P. vivax* was decreasing from 17.3% to 5.3% which shows that there was a trend of shift from *P. vivax* to *P. falciparum*, and mixed infection was less significant in the study area (1.5%) (Figure 1).

### 3.2. The Distribution of Malaria Cases with Age Groups

**Figure 2:**
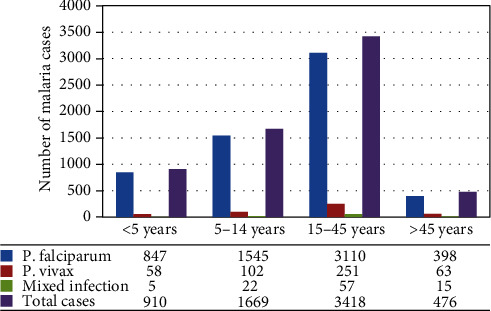
Distribution of malaria cases by age group in Nirak Health Center, Abergele District, Northeast Ethiopia.

Malaria case was reported in all age groups in the study area in the past five years. However, individuals in the age group between 15 and 45 years old contributed for the majority of cases with a prevalence rate of 52.8%, followed by 5-14 years old, less than 5 years old and greater than 45 years old with the prevalence rate of 25.8%, 14.0%, and 7.4%, respectively (Figure 2).

### 3.3. Monthly and Seasonal Patterns of Malaria over the Last Five Years

**Figure 3:**
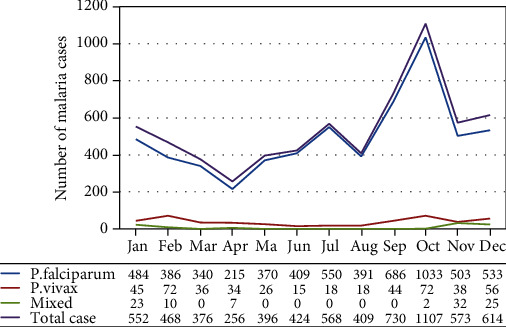
Distribution of malaria cases by month and Plasmodium species in Nirak Health Center, Abergele District, Northeast Ethiopia.

Over the last five years, malaria cases occurred in all months with a variable proportion. The highest proportion was reported in October with a slide positivity rate of 17.1%, followed by September, December, and November with a slide positivity rate of 11.3%, 9.5%, and 8.9%, respectively. On the other hand, the cases of malaria in April, March, and May were low when compared with other months with a slide positivity rates of 3.9%, 5.8%, and 6.1%, respectively. There was a fluctuated trend of *P. falciparum* and *P. vivax* throughout the years and months. The case report of *P. falciparum* was high in October, September, and December, whereas *P. vivax* was high in October, February, and December, months. Mixed cases were low in count and remained stable less fluctuate through each month (Figure 3).

Seasonal fluctuation is one of the elements that has a direct impact on malaria transmission. Despite the ups and downs, malaria cases were documented in every season of the year. In this study, malaria cases were high in fall or at the beginning of dry or “*Bega*” season; then followed by short rainy or “*Belg*” season and rainy summer or “*Keremit*” season with respective case burden of 55.2%, 23.1%, and 21.6%. *Plasmodium falciparum* infections followed the same seasonal pattern and showed steady increase from the beginning of short rainy season and continued to rise through summer reaching its peak during the fall or the beginning of dry season unlike *P. vivax*, in which the highest case was recorded in dry season followed by *Belg* (Table 2).

## 4. Discussion

Ethiopia envisages to eliminate malaria in accordance with global malaria elimination strategy by 2030. In line with this, Pilate elimination program has already been started in selected 239 districts in the country. Compilation of case reports, data organization, and interpretation of the information plays key role in measuring the impact of the ongoing elimination program. In this study of trend analysis, records of 19,433 malaria suspected patients were reviewed. Overall slide positivity rate of 33.3% was observed in the five-year period in Nirak Health Center. According to the findings of this study, malaria remains a severe public health problem in the area.

This result was comparable with the study done in Arjo-Didessa sugar development site (33.4%) and Walga Health Center (33.8%), Southern Ethiopia [14, 15] and Nakfa Hospital, Eritrea (33.0%) [16]. However, the finding was lower than the findings of studies conducted in Mankush Health Center, Western Ethiopia (51.04%) and Bale zone, Southeast Ethiopia (66.7%) [17, 18]. This disparity could be explained by differences in laboratory personnel's ability to correctly detect malaria parasites, mosquito control measures, population vulnerability, nearby water sources, community awareness of malaria parasite transmission and management, and malaria intervention practices.

This result was higher than similar studies conducted in Ataye, North Shoa, Ethiopia (8.4%), Woreta Health Center (5.4%), and Dembecha Health Center (16.34%), Northwest Ethiopia [19–21]. The variability of malaria prevalence might be due to differences in the implementation of malaria control and elimination strategies. Furthermore, the high prevalence of malaria in the study area might be due to climatic conditions. The district has an elevation of 1200 meters above sea level and average annual temperature and rainfall of 26°C and 786 ml, respectively. These climatic conditions favour the development and survival rates of both the *Anopheles mosquito* and the *Plasmodium* species. This greater temperature will also increase the frequency with which female adult mosquitoes feed, potentially increasing the risk of malaria transmission to uninfected human hosts [22].

The trend analysis results showed that malaria cases have been steadily growing over the last five years. The minimum and maximum numbers of cases were recorded in 2016 and 2020, respectively. According to a report from the district health office, the decline of malaria cases in 2016 was due to the severe problem of malaria prior to 2016, and there was a continuous drive to raise community knowledge about malaria prevention and control. Furthermore, all parties made every effort to keep the disease under control. The highest number of malaria cases reported in 2020 could be due to a lack of community understanding about malaria prevention and control strategies, increased number of nomads in the study area, the increment of irrigation activities in the health facility catchment areas, and failure to properly implement malaria control and elimination strategies. In addition, the likelihood of drug-resistance of malaria parasites in the study area and/or insecticide resistance of female anopheles mosquito populations might contribute to the highest number of malaria cases [23].

This nonfluctuating increasing trend of malaria cases was consistent with reports by a study conducted in Ataye, North Showa, Northcentral Ethiopia [19]. In contrast, studies conducted in Guba district, Benishangul-Gumuz regional state, Western Ethiopia [17], Dembecha Health Center [21], Bichena Primary Hospital, Amhara Region, Ethiopia [24], ultimately showed decline. This downward trend in those studies could be attributed to stakeholders' successive execution of malaria prevention and control actions in those locations to reduce malaria morbidity and mortality.

Regarding the plasmodium species distribution, about 91.2% and 7.3% of the malaria cases were due to *P. falciparum* and *P. vivax,* respectively. Mixed infection was found in 1.5% of malaria cases. This result was similar to that of a study conducted in Metema Hospital, Northwest Ethiopia [25]. However, it was disagreed with the national malaria parasite distribution pattern of Ethiopia [26], which showed that *P. falciparum* and *P. vivax* accounted for 60 and 40% of the malaria cases in the country, respectively. This variance could be due to the fact that this study was limited to a small malaria endemic area of the country, causing the prevalence of different species to vary. Furthermore, the study area's relatively lowland climatic conditions, where *P. falciparum* is a common species in the lowlands, as well as the possibility of treatment failure or recrudescence for *P. falciparum*, cannot be ruled out.

In comparison to previous years, *P. vivax* increased in 2019. This could be due to the parasite's dormant stage in the liver, which causes relapse, laboratory personnel's skill in identifying malaria species, and the emergence of chloroquine resistance [27]. In this study, males (62.2%) were more affected than females (37.8%). This was in agreement with the other previous studies [17, 28, 29]. This is because males are more likely to engage in outside activities, particularly agricultural ones, which puts them at risk of being bitten by infective anopheles' mosquitoes. Moreover, peak malaria transmission often occurs during the planting and harvesting seasons, and the majority of malaria cases in agricultural areas are among working adults [14, 21, 30].

Regarding the distribution of malaria prevalence by age group, the majority of reported cases were in the age group between 15 and 45 years. Such results have been reported by other studies [17, 21, 31]. In the study area, males in this age group spent most of their time, especially the evening outdoors to keep their domestic animals, crops, and other agricultural products. This could make the population more vulnerable to *Anopheles mosquito* bites.

The magnitude of malaria transmission depends on the community level of awareness, climatic, environmental, and seasonal factors. In this study, the highest cases of malaria were reported in October followed by September and December months. Likewise, the highest cases of malaria were observed during the beginning of dry season (September and October). This was in agreement with a national malaria indicator survey report made in 2016 [32]. The main malaria transmission season in most parts of Ethiopia is from September to December, following the rainy season from June to August [33]. The availability of mosquito vector breeding habitats, the duration of mosquito larvae development, and the rate of malaria parasite growth within the vector are all affected by seasonal variations in rainfall and temperature [34].

The second peak in malaria cases were documented during or towards the end of the “*Belg*” season. Other studies conducted in different parts of Ethiopia such as in West Gojam [21], Wolkite [35], and Arsi Negelle [36] also confirmed similar trends showing the bimodal peak nature of malaria transmission in the country with the first and bigger peak showing up in September-October and the second and minor peak in April to June. This is in contrast to some studies done in different parts of Ethiopia [17, 19, 36], which reported the second peak during the rainy season (June-August). This could be due to irrigation activities in the local area following harvesting in winter, which creates a favourable environment for mosquito breeding, which results in malaria outbreaks.

## 5. Conclusion

The prevalence of malaria is decreasing at the country level as a result of increased malaria prevention and control initiatives aimed at eliminating malaria in Ethiopia by 2030. However, the result of this trend analysis showed increased trends of malaria cases in Nirak Health Center, Abergele District, Northeast Ethiopia. This study found 33.3% overall slide positivity rate of malaria in the past five years in the study area. Malaria cases are increasing from time to time with the maximum cases recorded in 2020. This indicates that the national malaria control and elimination program should give close attention and strengthen the disease control strategies in this study area. *Plasmodium falciparum*, the causative agent of fatal or complicated forms of malaria, accounted 92.7% of all confirmed malaria cases in the study area. This could be a significant problem in achieving malaria elimination targets. Therefore, this finding alarms the local governments and other stack holders urgently to revise their intervention strategies, to give special attention and take action in this locality. Furthermore, researchers should do analytical studies to determine the cause of the high prevalence of *P. falciparum* in the studied area.

## Figures and Tables

**Table 1:** Suspected and slide-confirmed annual malaria cases at Nirak Health Center, Abergele District, Northeast Ethiopia.

Year	Number of examined *N* (%)	Slide-positive			Plasmodium species		
		Male *N* (%)	Female *N* (%)	Total cases *N* (%)	*P. falciparum* *N* (%)	*P. vivax* *N* (%)	Mixed *N* (%)
2016	2854 (14.7)	322 (71.2)	130 (28.8)	452 (15.8)	442 (97.8)	9 (2.0)	1 (0.2)
2017	2875 (14.8)	601 (66.3)	305 (33.7)	906 (31.5)	860 (94.9)	46 (5.1)	0 (0.0)
2018	4335 (22.3)	902 (59.4)	617 (40.6)	1519 (35.0)	1499 (98.7)	19 (1.2)	1 (0.1)
2019	4773 (24.6)	1112 (63.7)	635 (36.3)	1747 (36.6)	1348 (77.2)	302 (17.3)	97 (5.5)
2020	4596 (23.6)	1086 (58.7)	763 (41.3)	1849 (40.2)	1751 (94.7)	98 (5.3)	0 (0.0)
Total	19433 (100)	4023 (62.2)	2450 (37.8)	6473 (33.3)	5900 (91.2)	474 (7.3)	99 (1.5)

**Table 2:** Seasonal distribution of Plasmodium species in Nirak Health Center, Abergele District, Northeast Ethiopia.

Plasmodium species	Seasons		
	Dry season (September–January)	*Belg* season (February–May)	Rainy season (June–August)
*P. falciparum*	3239	1311	1350
*P. vivax*	255	168	51
Mixed	82	17	0
Total	3576	1496	1401

## Data Availability

All data are available within the manuscript. Additional data can be obtained from the corresponding authors up on request.
